# Medicinal Anti-Inflammatory Patch Loaded with Lavender Essential Oil

**DOI:** 10.3390/ijms25116171

**Published:** 2024-06-04

**Authors:** Karolina Zyburtowicz, Paulina Bednarczyk, Anna Nowak, Anna Muzykiewicz-Szymańska, Łukasz Kucharski, Aneta Wesołowska, Paula Ossowicz-Rupniewska

**Affiliations:** 1 Department of Chemical Organic Technology and Polymeric Materials, Faculty of Chemical Technology and Engineering, West Pomeranian University of Technology in Szczecin, Piastów Ave. 42, 71-065 Szczecin, Poland; 2 Department of Chemical and Process Engineering, Faculty of Chemical Technology and Engineering, West Pomeranian University of Technology in Szczecin, Piastów Ave. 42, 71-065 Szczecin, Poland; 3 Department of Cosmetic and Pharmaceutical Chemistry, Pomeranian Medical University in Szczecin, Powstańców Wielkopolskich Ave. 72, 70-111 Szczecin, Poland; 4 Department of Organic and Physical Chemistry, Faculty of Chemical Technology and Engineering, West Pomeranian University of Technology in Szczecin, Piastów Ave. 42, 71-065 Szczecin, Poland

**Keywords:** ibuprofen, *Lavandula angustifolia*, lavender essential oil, transdermal patch, cohesion, adhesion

## Abstract

Transdermal drug delivery offers a promising alternative for administering medications like ibuprofen, known for its analgesic and anti-inflammatory properties, with reduced gastrointestinal side effects compared to oral administration. This study explored the potential synergistic effects of combining ibuprofen with lavender essential oil (LEO) in transdermal patches. The composition of LEO was analyzed, revealing predominant compounds such as linalyl acetate and linalool, which are known for their analgesic and anti-inflammatory properties. The physicochemical properties of the patches were investigated, indicating improved cohesion with the addition of LEO. Additionally, thermal stability assessments demonstrated enhanced stability with LEO incorporation with an increase in onset decomposition temperature from 49.0 to 67.9 °C. The antioxidant activity of patches containing LEO was significantly higher with a free radical scavenging ability of 79.13% RSA compared to 60% RSA in patches without LEO. Release and permeation studies showed that patches with LEO exhibited an increased permeation of ibuprofen through the skin with 74.40% of the drug released from LEO-containing patches compared to 36.29% from patches without LEO after 24 h. Moreover, the permeation rate was notably faster with LEO, indicating quicker therapeutic effects. The inclusion of LEO in transdermal patches containing ibuprofen holds promise for enhancing drug delivery efficiency and therapeutic effectiveness, offering a potential strategy for improved pain management with reduced side effects.

## 1. Introduction

The transdermal delivery of medications such as ibuprofen is gaining increasing recognition for its potential to relieve pain while reducing the occurrence of side effects, especially when compared to the oral administration of nonsteroidal anti-inflammatory drugs (NSAIDs) [1,2,3,4,5,6]. Ibuprofen, an NSAID with analgesic, anti-inflammatory and antipyretic properties, is widely used in the treatment of various pain conditions, including rheumatic pain, menstrual pain, and post-traumatic pain. However, the traditional oral form of ibuprofen may be associated with the risk of gastrointestinal side effects, such as ulcers and bleeding, which limits its use in long-term therapy [7]. Transdermal drug delivery is a promising therapeutic strategy that enables efficient and convenient drug delivery through the skin. It has gained increasing interest in recent years due to the number of benefits it offers over traditional drug delivery methods such as oral administration or injections. Transdermal drug delivery avoids the first passage through the liver, which may reduce the risk of side effects and improve the bioavailability of the active substance. Additionally, it allows for constant, controlled distribution of the drug over a period of time, which may help maintain stable levels of the drug in the bloodstream and reduce the frequency of dosing [8,9,10,11,12]. The application on the skin is limited to some extent, and the main barrier limiting the permeation of drugs through the skin is the stratum corneum (SC), which prevents the entry of microorganisms and chemical substances as well as protects the organism against excessive water loss. The SC is a thin membrane consisting primarily of cornified epidermal cells, while the main intercellular components are ceramides and lipids [13,14]. In recent years, there has been a search for natural permeation enhancers of low toxicity while maintaining their enhancing activity [15]. Natural ingredients are often used to increase the penetration of certain substances through the skin, including terpenes found in essential oils. The dermatological products containing mainly “natural” ingredients are perceived by patients increasingly to be safer as compared to “synthetic” ingredients [16]. Furthermore, essential oils can generally be regarded as safe because they are rapidly metabolized, do not accumulate in the organism, and are quickly excreted after application to the skin [17,18]. One interesting essential oil that can be used as a permeation promoter is lavender oil. Lavender oil, obtained from the flowers of the *Lavandula angustifolia* plant, has been used for centuries for its various health properties, including analgesic, antioxidant, calming, anti-inflammatory effects, and antimicrobial activity [19,20,21,22,23,24,25,26]. Its analgesic effect is due to the content of compounds such as linalool and linalyl acetate, which have the ability to block the transmission of pain signals within the peripheral nervous system [27,28,29]. Moreover, previous studies have demonstrated the potential of lavender essential oil as an effective carrier for drug delivery, enhancing the permeation and therapeutic efficacy of various pharmaceuticals, such as asenapine maleate [30], penciclovir [31], lidocaine, caffeine [32], and p-aminobenzoic acid [18]. The use of essential oils such as clove, cinnamon, cyperus, chuanxiong, angelica, and turpentine [13,17,33] in increasing ibuprofen’s bioavailability is also known. Additionally, other studies have compared lavender essential oil and ibuprofen in attenuating stress-induced biochemical and behavioral changes in animal tests, demonstrating that both can significantly reduce stress markers. Lavender oil, in particular, showed more pronounced effects in diminishing stress-related alterations, indicating its potential as a more effective remedy for stress-related disorders compared to ibuprofen [34].

Interestingly, combining lavender oil with ibuprofen in transdermal patches may lead to a synergistic analgesic effect. Both ingredients, acting at different stages of pain signal transmission, can potentiate each other, leading to more effective pain relief at lower doses of drugs. In addition, lavender oil can also act as a skin penetration enhancer so that ibuprofen can be better and faster absorbed into the skin, which can increase the therapeutic effectiveness of medical patches containing this ingredient.

In this publication, medical patches containing ibuprofen and 5% lavender essential oil (LEO) were obtained. It presents how the addition of essential oil affects the release of ibuprofen from the drug form, permeability through the skin, and also the antioxidant properties and self-adhesive properties of obtained patches. This type of approach may not only increase the permeability of ibuprofen through the skin by using lavender oil as a permeation promoter but also create the possibility of potential synergism of both substances in pain relief.

## 2. Results and Discussion

### 2.1. Analysis of the Composition of Lavender Essential Oil

Table 1 showcases the identified components of *L. angustifolia* oils, their retention indices (RI), and relative percentage concentrations arranged according to their elution from an HP-5MS column. A total of 95 different constituents were identified in the commercially available lavender essential oil. Through GC-MS analysis, the primary constituents of the oils were determined to be linalyl acetate (34.90 ± 0.36%) and linalool (28.19 ± 0.09%). Additionally, notable components included camphor (3.55 ± 0.04%), β-caryophyllene (2.87 ± 0.16%), D-limonene (2.62 ± 0.03%), borneol (2.44 ± 0.02%), eucalyptol (2.41 ± 0.04%), terpinen-4-ol (2.32 ± 0.01%), and lavandulyl acetate (2.07–0.04%). The remaining ingredients were present in amounts below 2%. In our study, the main compounds of lavender oil were linalool and linalyl acetate, which is also confirmed by analyses carried out by other authors [35,36,37,38,39]. Both of these ingredients are monoterpene compounds with very wide biological activity when applied to the skin, among others, and they may have anti-inflammatory and antioxidant effects [28,40]. For example, linalool significantly prevented UVB-mediated 8-deoxy guanosine formation, causing oxidative DNA damage, as well as inhibited the UVB-induced phosphorylation of ERK1, JNK, and p38 proteins of the MAPK family. In addition, it may act as a photoprotective agent against UVB-induced skin damage [41]. Linalool and linalyl acetate also have an anesthetic effect because of their ability to block the transmission of pain signals within the peripheral nervous system [33,34], so they can be a very beneficial addition to pain patches applied to the skin.

### 2.2. Analysis of the Physicochemical Properties of the Obtained Transdermal Patches

**Figure 1:**
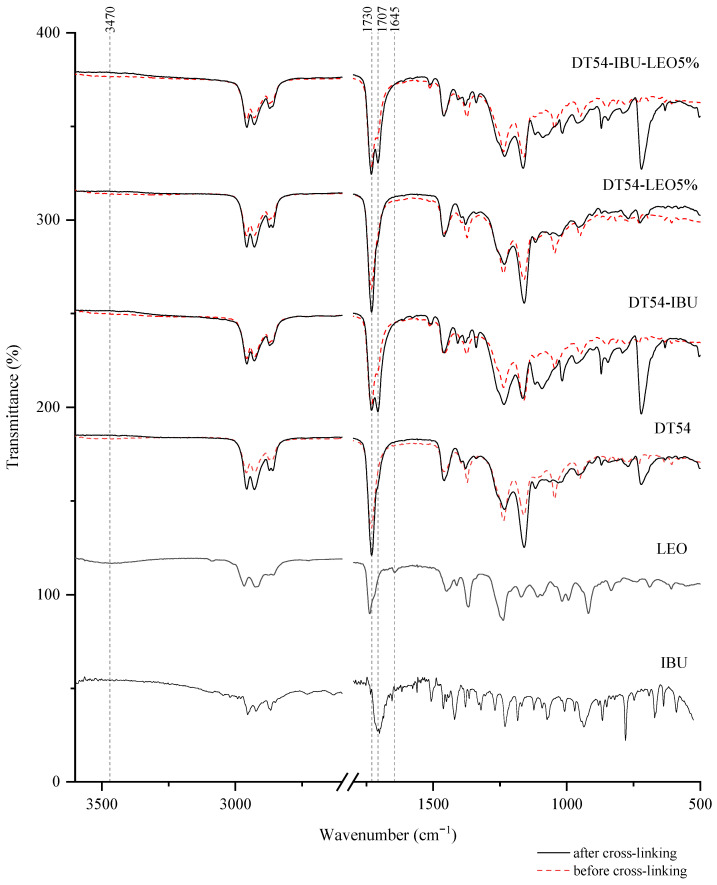
FTIR spectra of patch samples and the introduced active substances.

Interactions between the adhesive matrix and the introduced active substances were examined using infrared spectroscopy (FTIR). Figure 1 shows the FTIR spectra of ibuprofen (IBU), lavender essential oil (LEO), adhesive matrix patch (DT54), a patch in the form of an adhesive matrix with incorporated ibuprofen (DT54-IBU), a patch in the form of an adhesive matrix with a penetration promoter (DT54-LEO5%), and a patch with ibuprofen and a penetration promoter (DT54-IBU-LEO5%). Moreover, for a deeper analysis, the spectra of the patches before cross-linking are marked with a dashed line, and the spectra of the patches after cross-linking are marked with a solid line. All spectra of the obtained patches show peaks corresponding to the characteristics of the acrylate adhesive matrix, i.e., strong vibrations are present at 1730 cm^−1^ contributed by the -C=O of acrylate groups, and peaks that simultaneously occur in the adhesive matrix and the structure of ibuprofen, i.e., corresponding to the hydroxyl groups and hydrogen bonds in the range of 3000–2800 cm^−1^, and corresponding to the bonds stretching vibrations between C-O atom characteristics for ether (Ar-OR), ester (RCOOR’), and carboxylic acid (RCO-OH) groups, which are visible as bands at wavenumber 1235, 1163, 1093, 1015, and 966 cm^−1^. It can also distinguish the presence of a distinct peak at 1707 cm^−1^, corresponding to the bonds’ stretching vibrations between C=O atoms characteristic for carboxylic groups (RCO-OH), which is characteristic for patches containing ibuprofen in comparison to the patch without ibuprofen. It was also observed that the spectrum of LEO has peaks corresponding to hydroxyl groups at approximately 3470 cm^−1^ and unsaturated bonds at 1645 cm^−1^, which may ultimately affect the cross-linking of the adhesive matrix.

Table 2 shows the self-adhesive properties of the obtained transdermal patches. As expected, the presence of ibuprofen in the patch increased the coat weight. Moreover, the assessment of patches in terms of cohesion showed that the addition of IBU significantly reduces the effect of cohesive forces. However, it has been shown that the addition of lavender essential oil (LEO) improves the cohesion of patches with IBU. In the case of a small addition of LEO, the cohesion remains at a good level for use as transdermal patches. This is probably due to the presence of hydroxyl groups and/or unsaturated bonds of the main components of LEO, i.e., linalyl acetate (34.90 ± 0.36%) and linalool (28.19 ± 0.09%), which could be involved in the cross-linking of the adhesive matrix. Although the presence of the introduced modifiers reduced the adhesion, all the obtained patches were characterized by very good adhesion to the substrate, and values above 10 N 25 mm^−^^1^ are considered as such.

The thermal stability assessment of the acrylic pressure-sensitive adhesives and adhesive films from the obtained patches was conducted, and the results are summarized in Table 3. The parameters evaluated included temperature corresponding to 5% weight loss (T_d_^5%^), temperature corresponding to 50% weight loss (T_d_^50%^), maximum decomposition temperature (T_MDT_), and glass transition temperature (T_g_). For the acrylic pressure-sensitive adhesives before cross-linking, the onset decomposition temperature (Td5%) ranged from 49.0 °C for DT54-LEO5% to 67.9 °C for DT54-IBU, the temperature corresponding to 50% weight loss (T_d_^50%^) ranged from 321.6 °C for DT54-IBU to 356.0 °C for DT54-LEO5%, and the maximum decomposition temperature (TMDT) ranged from 357.1 °C for DT54-IBU-LEO5% to 368.9 °C for DT54-LEO5%. Upon cross-linking to form adhesive films, the T_d_^5%^, T_d_^50%^, and T_MDT_ values differed. Notably, the T_d_^5%^ ranged from 180.8 °C for DT54-IBU-LEO5% to 291.8 °C for DT54-LEO5%, the T_d_^50%^ ranged from 344.9 °C for DT54-IBU to 367.1° C for DT54, and the TMDT ranged from 336.9 °C for DT54-IBU to 372.2 °C for DT54-IBU-LEO5%. The results indicate variations in thermal stability between adhesive formulations with differences observed between adhesive patches with and without the incorporation of ibuprofen and penetration promoters. Generally, the adhesive layer without the active substance exhibited higher stability compared to those with ibuprofen or penetration promoters. This aligns with previous research findings. The onset of degradation for the commercial adhesive DT54 was notably higher compared to DT54-IBU, indicating the influence of the active substance on stability. Surprisingly, the addition of volatile essential oil has a beneficial effect on the thermal stability of the obtained adhesive film. Additionally, the temperatures corresponding to 50% weight loss and the maximum decomposition temperatures were similar across adhesive formulations with most adhesives exhibiting values higher than those corresponding to 50% weight loss. The inverse relationship was observed in the case of cross-linked adhesive films.

**Figure 2:**
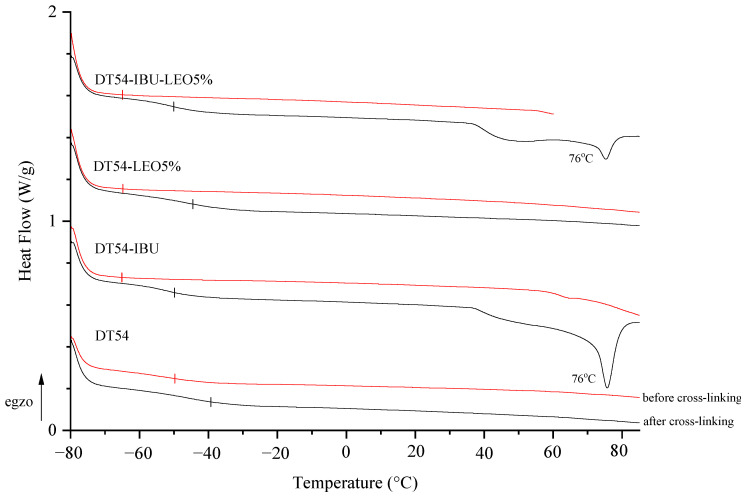
DSC thermograms of the obtained adhesive compositions (before cross-linking) and patches (after cross-linking).

The adhesive matrix composition before cross-linking (DT54) has a glass transition temperature (T_g_) of −50 °C. In turn, adhesive compositions with IBU or LEO additives before cross-linking are characterized by a similar glass transition temperature, which is −65 °C. Cross-linking the samples resulted in a higher glass transition temperature and a different temperature in the case of DT54-LEO5% (−45 °C) compared to other patches with additives (−50 °C). Unfortunately, the introduction of the drug causes an excessive reduction in the T_g_ of the patches, which results in low cohesion of the adhesive films, but this is within the acceptable range for this type of application. Additionally, in the thermograms of cross-linked IBU patches (both DT54-IBU and DT54-IBU-LEO5%), a clear peak at 76 °C corresponding to the melting of the drug used can be seen (Figure 2).

### 2.3. Antioxidant Activity

Our study also assessed the antioxidant activity of the analyzed patches. For comparison, the antioxidant activity of the LEO used to prepare DT54-IBU-LEO5% was determined. The results of the percentage of free radical scavenging capacity are presented in Table 4. The patch with the addition of essential oil showed significantly higher antioxidant activity compared to the patch without the oil. The essential oil added to the patch had a very high free radical scavenging ability of 79.13% RSA, which is undoubtedly reflected in the antioxidant activity of the patch prepared with it. The antioxidant activity of essential oil has been confirmed by many previous authors [42,43,44,45]. The antioxidant activity of topically applied patches may play an important role in eliminating, for example, bacterial infections. Bacterial infections located in the skin and underlying tissues may be caused, among other things, by excessive oxidative stress [46]. For example, *S. aureus* infection induces reactive oxygen species (ROS) in neutrophils, macrophages, and leukocytes; as a consequence, it increases free radical production and reduces the antioxidant response of these cells [47,48]. In addition, more ROS is released during inflammation, which protects the body against microorganisms [49]. Therefore, adding topically applied lavender oil to a patch may increase its pharmacological effect through antioxidant and antibacterial properties.

### 2.4. Release and Permeation Study

**Figure 3:**
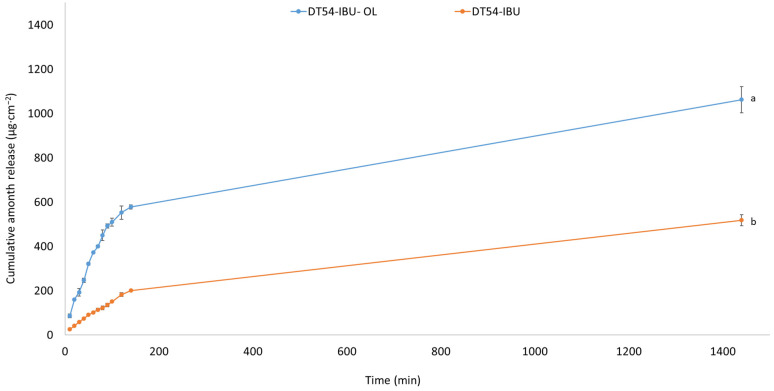
Time course of the IBU during the 24 h release *p* < 0.001, α = 0.05, mean ± SD, n = 3. The statistically significant difference was estimated by ANOVA using Tukey’s test. Different letters indicate important differences between obtained patches.

In our study, the release of the active compound from the medical patch was determined, as shown in Figure 3. The highest amounts of IBU are released in the first 3 h, especially for the DT54-IBU-LEO5%, and after this time, IBU is inhibited. After 24 h of release, 74.40% of the drug was released from the DT54-IBU-LEO5%, which was statistically significant compared to the DT54-IBU—36.29%. The essential oils used in the patch do not limit the number of active ingredients released.

The permeability parameters of two different patch samples, DT54-IBU and DT54-IBU-LEO5%, were evaluated, and the results are presented in Table 5. The cumulative permeation mass, flux (J_SS_), apparent permeability coefficient (K_P_), and the percentage of drug that penetrated after 24 h (Q%_24 h_) were measured. Comparing the results, DT54-IBU-LEO5% showed higher values across all parameters compared to DT54-IBU. Specifically, DT54-IBU-LEO5% exhibited a significantly higher cumulative permeation mass, flux, and apparent permeability coefficient. For DT54-IBU, there was a corresponding flux of 9.292 ± 1.891 µg cm^−2^∙h^−1^ and an evident permeability coefficient (K_P_) of 6.504 ± 1.323 cm h^−1^. Additionally, the percentage of ibuprofen that penetrated after 24 h was determined to be 4.09 ± 0.26%. In comparison, DT54-IBU-LEO5% exhibited higher values across all parameters. The flux was notably increased to 11.921 ± 3.481 µg cm^−2^∙h^−1^, which was accompanied by a greater apparent permeability coefficient of 8.344 ± 2.436 cm h^−1^. Furthermore, the percentage of ibuprofen that penetrated after 24 h significantly rose to 9.169 ± 0.57%. This indicates that the inclusion of LEO5% as an enhancer led to enhanced permeation of the active substance through the skin. These results align with the broader study findings, which indicated that the use of enhancers resulted in higher levels of active substance permeation after 24 h [2], and the essential oil can be treated as a penetration enhancer. The variations in permeation observed among patches containing different enhancers underscore the impact of enhancer selection on permeability outcomes.

**Figure 4:**
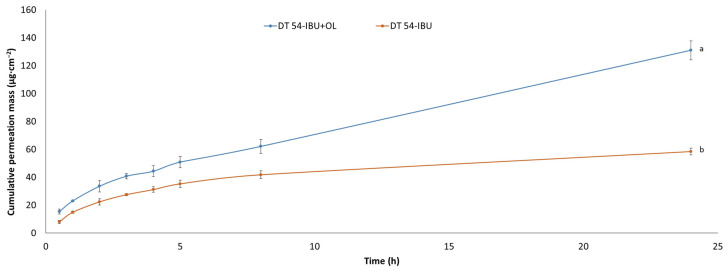
The IBU permeation profiles. Values are the means with standard deviation: *p* < 0.001, α = 0.05, mean ± SD, n = 3. The statistically significant difference was estimated by ANOVA using Tukey’s test. Different letters are important differences between obtained patches.

Our study also examined the permeation through the skin of IBU under the influence of added essential oil. Figure 4 shows the penetration of ibuprofen from the analyzed patches. The use of essential oil significantly increases the flux of IBU. The accumulated mass of IBU in the acceptor chamber after a 24 h test after the application of DT54-IBU-LEO5% was significantly higher (131.00 ± 6.82 µg·cm^−2^) compared to the DT54-IBU (58.44 ± 2.41 µg·cm^−2^). Many authors report an increased penetration of drugs through the skin after the additional use of essential oils. For example, the *Eugenia caryophyllata* essential oil also increased the penetration of ibuprofen through the skin [17,50]. In previous studies, it was also found that essential oils increased the delivery to the skin of other substances, such as vitamins [51], 5-fluorouracil, indomethacin [52], and chlorhexidine digluconate [53]. In another study, the penetration of flurbiprofen from the patch through the skin of rats increased significantly after adding 5% (*v*/*v*) of the *Ocimum sanctum* essential oil and turpentine oil to it [54]. It is known that the components of essential oils that increase penetration are the terpenes present in them. In our study, in LEO, the largest amounts of linalyl acetate and linalool were identified. Numerous studies report that linalool is a terpene that increases the penetration of active substances through the skin [55,56]. The mechanism of increasing penetration by terpenes contained in essential oils is primarily through interaction with SC lipids and by increasing the solubility of the drug into SC lipids [57,58]. In the case of lipophilic drugs, terpenes increase the stratum corneum/vehicle partition coefficient. The penetration of lipophilic drugs increases in proportion to their solubility in the enhancer or enhancer solution [58].

**Figure 5:**
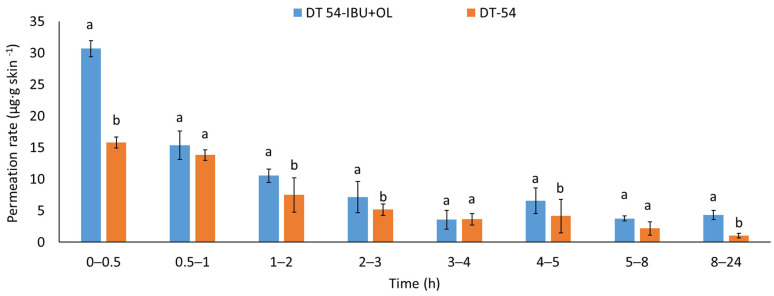
The permeation rate of IBU during the 24 h permeation; α = 0.05 *p* < 0.001, α = 0.05, mean ± SD, n = 3. The statistically significant difference was estimated by ANOVA using Tukey’s test. Different letters indicate important differences between obtained patches.

The permeation rate was also determined by measuring the therapeutic effect obtained. When applying patches containing anti-inflammatory and analgesic substances to the skin, it is very important that these substances quickly penetrate into the deeper layers of the skin to show an immediate therapeutic effect. Figure 5 shows the permeation rate for each time interval. The highest permeation rate for both patches was obtained in the first hours of the measurement, especially in 0.5–2 h. Added LEO significantly increases the permeated rate of the active compound. The penetration of ibuprofen from this patch was much faster in the first hour after applying the patch to the skin.

**Figure 6:**
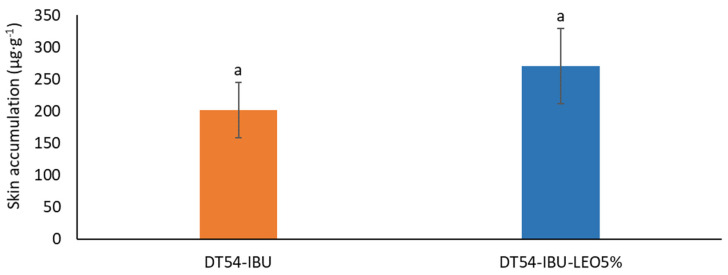
The skin accumulation of IBU after 24 h permeation. *p* < 0.001, α = 0.05, mean ± SD, n = 3. The statistically significant difference was estimated by ANOVA using Tukey’s test. Different letters indicate important differences between obtained patches.

Figure 6 shows the mass of IBU accumulated in porcine skin after 24 h of penetration. A slightly greater accumulation of ibuprofen in the skin was observed after applying the patch with essential oil but without significant statistical differences compared to the DT54-IBU-LEO5%. The slightly greater accumulation was probably due to the influence of LEO, whose terpenes loosened the SC, thus causing greater penetration of the drug.

## 3. Materials and Methods

### 3.1. Materials

A transdermal patch utilizing a commercial polyacrylate adhesive, specifically DURO-TAK 378-2054 (DT54), was formulated. This adhesive, categorized as a drug-in-adhesives matrix type, exhibits a viscosity of 1.46 Pa·s and a solid weight concentration (SWC) of 49.7%. Its composition includes an acrylate copolymer blended with solvents such as propan-2-ol (10–20%), ethyl acetate (10–20%), n-heptane (1–5%), petroleum (1–5%), methylcyclohexene (1–5%), and toluene (1–3%). The copolymer is derived from the copolymerization of 2-ethylhexyl acrylate, acrylic acid, butyl acrylate, and vinyl acetate. Additional components in the reaction mixture consist of aluminum tris(2,4-pentanedionato-O,O′), pentane-2,4-dione, and azobisisobutyronitrile (AIBN).

Ibuprofen (99%) (IBU), sourced from Sigma Aldrich (Steinheim am Albuch, Germany), was incorporated into the transdermal patches as an active ingredient.

This research used commercial natural oil obtained from fresh lavender flower heads (Etja, Elbląg, Poland).

Additional reagents employed in the permeation tests included PBS buffer pH 7.4 sourced from Merck (Darmstadt, Germany), high-purity orthophosphoric acid (98%) obtained from Chempur (Piekary Śląskie, Poland), HPLC gradient grade acetonitrile (≥99.9%) and methanol (99.9%) provided by Sigma-Aldrich (Steinheim am Albuch, Germany), and anhydrous potassium dihydrogen phosphate (99%) (KH2PO4) supplied by Merck (Darmstadt, Germany).

### 3.2. Analysis of the Composition of Lavender Essential Oil

A Hewlett Packard 6890 gas chromatograph coupled with a Hewlett Packard 5973 Mass Selective Detector and 6890 Series Injector was used to examine the chemical composition of lavender essential oil. The setup included an HP-5MS capillary column (30 × 0.25 mm i.d., with a film thickness of 0.25 μm), and helium served as the carrier gas at a flow rate of 1.2 cm^3^ min^−1^. The operating parameters were set as follows: injector temperature at 250 °C, transfer line temperature at 280 °C, and ion source temperature at 230 °C. The GC oven temperature followed a programmed sequence: starting at 45 °C, it increased to 200 °C at a rate of 5 °C min^−1^ (maintained for 10 min); then, it ramped up to a final temperature of 250 °C at a rate of 5 °C min^−1^ (maintained for 20 min). For each analysis, 1 μL of the sample (equivalent to 20 mg of essential oil dissolved in 1.5 cm^3^ of dichloromethane) was injected using a split ratio of 5:1. Mass spectra were acquired at 70 eV (EI). They scanned (2.94 scans/s) within the range of 50–550 *m*/*z*. A single sample analysis lasted 71 min. Compound identification relied on a comparison of mass spectra with entries in MS databases (Wiley, Hoboken, NJ, USA, NIST 2002) as well as with authentic compounds available in our laboratory (e.g., β-pinene, p-cymene, limonene, camphor, menthone, carvone, carvacrol, thymol). Further confirmation was obtained by comparing calculated retention indices (relative to n-alkanes: C7-C30, from Supelco, Bellefonte, PA, USA, using an HP-5MS column) with data from the NIST Chemistry WebBook (https://webbook.nist.gov/chemistry/ (accessed on 10 May 2024) and literature references [59]. Relative percentages of essential oil constituents were determined from the total peak area (TIC) using MSD Enhanced ChemStation G1701AA ChemStation A.03.00. software. 

### 3.3. Preparation of Adhesive Films

The adhesive matrix for the transdermal patches was composed of a commercial acrylate copolymer. Initially, a patch consisting solely of the adhesive matrix (DT54) was prepared, which was devoid of IBU and penetration promoters. Subsequently, patches were formulated with IBU (DT54-IBU) and patches containing both IBU and 5% lavender essential oil (DT54-IBU-LEO5%) and 5% lavender essential oil (DT54-LEO5%). The weight ratio of the adhesive matrix to the active substance was determined based on the adhesive properties, including solids content, as well as the characteristics of the active substance, such as molar mass, and an initial assumption regarding the active substance content in commercial products, i.e., 200 mg of ibuprofen for a surface area of the adhesive film equal to 140 cm^2^. For the adhesive compositions in this series, the active substance was dissolved in ethyl acetate and then added to the adhesive matrix with or without lavender essential oil. Subsequently, the adhesive compositions were coated (250 µm) onto a polyester film. The resulting polymer layers were then thermally cross-linked for 15 min at 110 °C. Finally, the adhesive film layer was covered with siliconized release paper. Table 6 displays the appropriate weights for preparing the formulations.

### 3.4. Coat Weight of Cross-Linked Adhesive Films

To assess the coat weight of the cross-linked adhesive films post-solvent evaporation, a circular punch with a 10 cm^2^ area (Karl Schröder KG, Weinheim, Germany) was employed.

### 3.5. Self-Adhesive Properties of Cross-Linked Adhesive Films

The self-adhesive properties of the coated, cross-linked adhesive were evaluated, including tack, adhesion, and cohesion at 20 and 70 °C, following international standards AFERA and FINAT protocols. Shear strength was assessed in accordance with FINAT FTM 8, adhesion according to AFERA 4001, and tack according to AFERA 4015. Testing was conducted using a Zwick/Roell Z-25 testing machine.

Cohesion was determined as the time it took for the adhesive film to separate from the steel plate under a one-kilogram load, as per standard [60].

Peel adhesion, reflecting the force needed to remove a coated flexible pressure-sensitive adhesive sheet material from a test panel, was evaluated using the Zwick/Roell Z010 testing machine at room temperature. Adhesive films measuring 2.5 × 12.7 cm were prepared after cross-linking onto a standard steel plate. After 20 min, the plate was placed in the lower holder of the testing machine with the second part positioned horizontally on the steel plate. The test involved peeling the adhesive tape at a 180° angle with a jaw speed of 300 mm min^−1^, measuring the force required to detach the adhesive film from the steel plate [60].

### 3.6. Thermal Stability and Phase Transition Temperatures of Adhesives and Cross-Linked Adhesive Films

Thermal stability was evaluated using a thermogravimetric analysis performed with a TG 209 F1 Libra thermomicrobalance from Netzsch. Samples weighing approximately 10 mg underwent heating at a rate of 10 °C min^−1^ in an atmosphere containing nitrogen (as a protective gas at 10 cm^3^ min^−1^) and air (at 25 cm^3^ min^−1^) across a temperature range of 25 to 1000 °C. The onset decomposition temperature was determined by analyzing the intersection of tangents on the TG curve. In contrast, the temperatures corresponding to the most significant weight loss were identified from the first derivative of the TG curve (DTG curve).

For assessing the glass transition temperature of the adhesive, differential scanning calorimetry (DSC) analysis was conducted using a Q-100 differential calorimeter from TA Instruments, which is located in New Castle, DE, USA (2004). Samples underwent a heating cycle from −80 to +100 °C at a heating rate of 10 °C min^−1^.

### 3.7. Antioxidant Activity of Patches and L. angustifolia Essential Oil

The scavenging activity of DPPH stable free radicals was measured as described previously [16,61]. In the case of patches, the diffusion surfaces (1 cm^2^) were extracted in ethanol for 24 h. Next, samples of 0.15 cm^3^ collected of extract as well as LEO were mixed with 2.85 cm^3^ of 0.3 mM DPPH radical solution dissolved in 96% *v*/*v* ethanol. The absorbance at 517 nm of the DPPH working solution was adjusted to 1.00 ± 0.02 with 96% (*v*/*v*) ethanol. Measurement of absorbance at 517 nm against 70% (*v*/*v*) ethanol was performed after 10 min of incubation in the dark at room temperature. Three independent samples of each examined extract were prepared.

As a result, seven measurement points were realized for each concentration of the tested extract after 24 h of incubation of patches or LEO. The scavenging activity on the DPPH radical was expressed as an inhibition percentage using the following equation:

$$\%DPPH\cdot scavenging=1-\frac{As}{Ac}\cdot 100\%$$

where

As—absorbance of the tested sample;
Ac—absorbance of the control sample.

Measurements were carried out in triplicate for each sample of extract.

### 3.8. In Vitro Skin Permeation, Release, and Accumulation Studies

Franz diffusion cells (Phoenix DB-6, ABL&E-JASCO, Wien, Austria) with a diffusion area of 1 cm^2^ were utilized for the permeation experiments. The acceptor chamber, equipped with a magnetic stirring bar, contained 10 cm^3^ of pH 7.4 PBS solution to maintain constant conditions, including a temperature of 37.0 ± 0.5 °C and consistent stirring. Porcine skin, chosen for its similarity in permeability to human skin, was fresh from a local slaughterhouse. The skin was washed in PBS buffer pH 7.4 and dermatomed to a thickness of 0.5 mm. The skin was stored in aluminum foil at −20 °C for three months before experimentation to preserve its barrier properties. Prior to use, the skin samples were thawed slowly at room temperature for 30 min and hydrated with PBS pH 7.4. Only samples with an impedance >3 kΩ, akin to human skin, were employed.

Patches were applied to the skin, and as previously described, the experiment ran for 24 h with samples collected at specified intervals. Release tests followed the same procedure as permeation tests but with a membrane covering the diffusion areas. A dialysis tubing cellulose membrane (D 9777-100FT, Sigma Aldrich, Steinheim am Albuch, Germany) covered the diffusion areas, and the prepared patch was placed over it. The acceptor cell contained 10 cm^3^ of pH 7.4 PBS, maintained at 37 °C, and the experiment spanned 24 h. The solubility of ibuprofen in a PBS solution with pH 7.4 is 0.432 ± 0.001 g dm^−3^. Samples were taken at specified intervals, and 0.4 cm^3^ of acceptor fluid aliquots were withdrawn and replaced with fresh buffers of the same pH. Compound concentrations in the acceptor fluid were measured using HPLC.

The accumulation of the tested substance in the skin post-penetration was determined using established methods. The supernatant was collected and analyzed using HPLC. The accumulation of IBU in the skin was calculated as the ratio of the drug remaining in the skin to the mass of the skin sample, which is expressed as μg g^−1^. A liquid chromatography system (Knauer, Berlin, Germany) assessed IBU and its derivatives in the acceptor fluid during permeation and release tests and accumulation in the skin.

The HPLC system comprised a model 2600 UV detector, a Smartline model 1050 pump, and a Smartline model 3950 autosampler with ClarityChrom V2.6. 2009 software (Knauer, Berlin, Germany). The detector operated at 264 nm, and a chromatographic column measuring 125 × 4 mm filled with Hypersil ODS (C18), with a particle size of 5 µm, was employed. The mobile phase consisted of 0.02 M potassium dihydrogen phosphate–acetonitrile–methanol (45/45/10 *v*/*v*) with a flow rate of 1 cm^3^ min^−1^. The column temperature was maintained at 25 °C, and the injection volume was 20 μL.

Concentrations of IBU in the acceptor phase were determined by HPLC, and cumulative mass (µg cm^−2^) was calculated based on this concentration. Flux (µg cm^−2^ h^−1^) through pigskin into the acceptor fluid was determined as the slope of the cumulative mass plot against time.

### 3.9. Statistical Analysis

Results are presented as the mean ± standard deviation (SD). One-way analysis of variance (ANOVA) was conducted for statistical analysis, and Tukey’s test (α < 0.05) was applied to evaluate the significance of differences between individual groups in cumulative mass. Statistical computations were performed using Statistica 13.3 PL software (StatSoft, Kraków, Poland).

## 4. Conclusions

In conclusion, the addition of *Lavendula angustifolia* essential oil to patches demonstrated superior permeation properties on pig skin compared to the pure patch. This enhancement suggests that adding essential oil to the patch may increase its effectiveness in delivering the active compound. Furthermore, incorporating LEO into the patch may enhance its antioxidant properties, potentially offering additional therapeutic benefits. Importantly, the addition of LEO did not deteriorate the physicochemical properties of the adhesive matrix, including the self-adhesive properties of the obtained patches. These findings highlight the potential of lavender oil as a natural enhancer in transdermal drug delivery systems, offering improved therapeutic outcomes without compromising patch integrity.

## Figures and Tables

**Table 1:** Determined compounds and their percentage composition in the analyzed lavender essential oil.

No.	Compound	Rt [min]	RI_exp_.	RI_Lit_.	Relative Content [%]	SD
1.	(E)-Hex-2-en-1-ol	4.72	852	853	0.03	0.01
2.	(Z)-Hex-2-en-1-ol	4.96	865	866	0.08	0.01
3.	2-Heptanone	5.44	889	889	0.05	0.00
4.	Tricyclene	6.20	922	921	0.09	0.01
5.	α-Thujen	6.31	926	927	0.09	0.01
6.	α-Pinene	6.48	933	934	0.33	0.01
7.	Camphene	6.85	947	949	1.06	0.02
8.	β-Thujene	7.49	973	973	0.08	0.01
9.	β-Pinene	7.57	976	977	0.31	0.01
10.	1-Octen-3-ol	7.81	985	986	0.40	0.00
11.	3-Octanone	7.93	990	991	0.39	0.03
12.	3-Octanol	8.05	995	994	0.14	0.00
13.	α-Phelandrene	8.29	1004	1003	0.02	0.03
14.	3-Carene	8.46	1010	1009	0.14	0.01
15.	Hexyl acetate	8.53	1013	1014	0.27	0.00
16.	α-Terpinene	8.63	1016	1016	0.03	0.06
17.	m-Cymen	8.78	1022	1023	0.05	0.00
18.	p-Cymen	8.85	1024	1025	0.55	0.07
19.	D-Limonene	8.97	1028	1029	2.62	0.03
20.	Eucalyptol	9.04	1031	1031	2.41	0.04
21.	(Z)-β-Ocymene	9.21	1037	1038	1.25	0.34
22.	(E)-β-Ocimene	9.49	1047	1049	0.60	0.22
23.	γ-Terpinene	9.80	1058	1059	0.08	0.06
24.	cis-Sabinene hydrate	10.03	1066	1066	0.10	0.01
25.	Cis-linalool oxide	10.19	1072	1073	0.19	0.01
26.	Trans-linalool oxide	10.64	1088	1088	0.31	0.03
27.	Linalool	11.11	1105	1105	28.19	0.09
28.	Nonanal	11.16	1107	1107	0.08	0.10
29.	1-Octen-3-yl acetate	11.31	1112	1111	0.45	0.01
30.	Fenchol	11.41	1116	1117	0.02	0.02
31.	(Z)-p-Menth-2-en-1-ol	11.59	1122	1122	0.02	0.03
32.	β-Thujone	11.63	1123	1124	0.12	0.02
33.	α-Campholenal	11.78	1129	1130	0.02	0.02
34.	allo-Ocimene	11.90	1133	1131	0.22	0.01
35.	(E)-Pinocarveol	12.06	1139	1140	0.05	0.02
36.	cis-Sabinol	12.14	1142	1143	0.03	0.01
37.	Camphor	12.25	1146	1145	3.55	0.04
38.	(Z)-2-Nonenal	12.31	1148	1148	0.02	0.05
39.	Nerol oxide	12.58	1157	1155	0.20	0.01
40.	Borneol	12.84	1167	1168	2.44	0.02
41.	1-Nonanol	13.04	1174	1175	0.44	0.01
42.	Terpinen-4-ol	13.17	1178	1178	2.32	0.01
43.	p-Cymen-8-ol	13.28	1182	1183	0.06	0.01
44.	Cryptone	13.41	1187	1187	0.22	0.02
45.	α-Terpineol	13.53	1191	1192	1.73	0.04
46.	Myrtenal	13.71	1198	1197	0.17	0.03
47.	Dodecan	13.81	1201	1200	0.04	0.04
48.	Verbenone	14.06	1210	1211	0.05	0.03
49.	(Z)-Carveol	14.57	1229	1229	0.28	0.02
50.	Pulegone	14.77	1236	1237	0.16	0.01
51.	Cuminal	14.91	1241	1240	0.19	0.01
52.	D-Carvone	15.00	1245	1246	0.04	0.01
53.	Linalyl acetate	15.45	1261	1259	34.90	0.36
54.	trans-Geraniol	15.58	1266	1269	0.07	0.01
55.	α-Citral	15.73	1271	1271	0.04	0.04
56.	Phellandral	15.77	1273	1274	0.13	0.02
57.	Citronellol formate	15.93	1279	1278	0.02	0.02
58.	Neryl formate	15.98	1281	1284	0.20	0.02
59.	Borneol acetate	16.16	1287	1288	0.18	0.02
60.	Lavandulyl acetate	16.25	1290	1291	2.07	0.04
61.	Carvacrol	16.48	1299	1299	0.03	0.03
62.	Bicycloelemene	17.29	1330	1331	0.12	0.01
63.	Linalyl propionate	17.38	1333	1333	0.02	0.01
64.	δ-Elemene	17.51	1338	1339	0.09	0.03
65.	Piperitenone	17.60	1342	1343	0.03	0.03
66.	α-Cubebene	17.81	1350	1351	0.53	0.04
67.	Thymol acetate	17.93	1355	1355	0.10	0.05
68.	Neryl acetate	18.17	1364	1364	0.36	0.01
69.	Capric acid	18.46	1375	1374	0.03	0.01
70.	3-Methyltridecane	18.54	1378	1377	0.03	0.01
71.	Geranyl acetate	18.67	1383	1384	0.79	0.01
72.	Geranyl acetate	18.73	1385	1385	0.07	0.00
73.	β-Cubenene	18.79	1388	1388	0.03	0.02
74.	β-Bourbonene	18.88	1391	1390	0.08	0.01
75.	Longifolene	19.27	1406	1407	0.07	0.01
76.	Dodecanal	19.35	1410	1410	0.02	0.00
77.	cis-α-Bergamotene	19.52	1417	1416	0.06	0.00
78.	β-Caryophyllene	19.68	1423	1423	2.87	0.16
79.	trans-α-Bergamotene	20.03	1437	1438	0.45	0.03
80.	Aromadendrene	20.28	1447	1447	0.04	0.00
81.	(E)-β-Farnesene	20.52	1457	1458	1.25	0.10
82.	Alloalomadendrene	20.60	1460	1462	0.03	0.01
83.	α-Humulene	20.71	1465	1465	0.08	0.01
84.	γ-Muurolene	21.20	1484	1485	0.36	0.14
85.	β-Selinene	21.26	1487	1486	0.02	0.02
86.	Bicyclogermacrene	21.63	1502	1503	0.06	0.01
87.	β-Bisabolen	21.81	1509	1509	0.31	0.02
88.	γ-Cadinene	21.98	1517	1517	0.26	0.01
89.	β-Sesquiphellandrene	22.08	1521	1522	0.02	0.00
90.	δ-Cadinene	22.18	1525	1524	0.10	0.02
91.	Germacrene B	22.90	1556	1557	0.06	0.02
92.	Caryophyllene oxide	23.63	1587	1589	0.64	0.20
93.	Humulene epoxide II	24.35	1618	1619	0.02	0.00
94.	α-Muurolol	24.91	1643	1645	0.18	0.01
95.	α-Bisabolol	25.83	1685	1683	0.16	0.02
Total identified [%]					99.70	

Rt—retention time; RI_exp._—retention indices relative to n-alkanes (C_7_–C_30_) on HP-5 MS capillary column; RI_Lit._—literature retention indices.

**Table 2:** Self-adhesive properties of the obtained transdermal patches.

Sample Name	Coat Weight [g/m^2^]	Cohesion		Adhesion [N/25 mm]
		20 °C	70 °C	
DT54	73	>72 h (c.f)	>72 h (c.f)	27.2
DT54-IBU	92	22 s (c.f)	48 s (c.f)	22.0
DT54-LEO5%	66	31 h 20 min (c.f)	40 min (c.f)	22.7
DT54-IBU-LEO5%	82	11 s (c.f)	5 s (c.f)	19.0

**Table 3:** The thermal stability assessment and glass transition temperatures of acrylic pressure-sensitive adhesives and adhesive films from obtained patches.

Sample Name	T_d_^5%^ (°C)	T_d_^50%^ (°C)	T_MDT_ (°C)	T_g_ (°C)
Acrylic pressure-sensitive adhesives (before cross-linking)				
DT54	52.3	355.1	366.5	−50
DT54-IBU	67.9	321.6	365.4	−65
DT54-LEO5%	49.0	356.0	368.9	−65
DT54-IBU-LEO5%	63.6	329.0	357.1	−65
Adhesive films from obtained patches (after cross-linking)				
DT54	287.3	367.1	364.6	−40
DT54-IBU	190.5	344.9	336.9	−50
DT54-LEO5%	291.8	364.1	366.3	−45
DT54-IBU-LEO5%	180.8	354.0	372.2	−50

T_d_^5%^—5% weight loss temperature, T_d_^50%^—50% weight loss temperature, T_MDT_—maximum decomposition temperature, T_g_—glass transition temperature.

**Table 4:** The antioxidant activity of patches and pure *L. angustifolia* essential oil was measured using the DPPH method. *p* < 0.001, α = 0.05, mean ± SD, n = 3. Different letters indicate significant differences between obtained patches. The statistically significant difference was estimated by ANOVA using Tukey’s test. LEO—*L*. *angustifolia* essential oil.

Sample Name	% RSA
DT54-IBU	1.22 ± 0.28 ^a^
DT54-IBU-LEO5%	11.50 ± 0.40 ^b^
LEO	79.13 ± 0.98

**Table 5:** Skin permeation parameters for ibuprofen, mean ± SD, and n = 3.

Sample Name	Cumulative Permeation Mass [µg cm^−2^]	J_SS_ [µg cm^−2^ h]	K_P_ [cm h^−1^]	Q%_24 h_
DT54-IBU	58.435 ± 3.689	9.292 ± 1.891	6.504 ± 1.323	4.09 ± 0.26
DT54-IBU-LEO5%	130.998 ± 8.088	11.921 ± 3.481	8.344 ± 2.436	9.169 ± 0.57

J_SS_—steady-state flux; K_P_—permeability coefficient; Q%_24 h_—per cent drug permeated after 24 h.

**Table 6:** Weight ratios of individual ingredients contained in the adhesive composition.

Sample Name	Adhesive Mass [g]	Ibuprofen Mass [g]	Lavender Essential Oil Mass [g]	Solvent Mass [g]
DT54	6.765	-	-	1.5
DT54-IBU	6.765	1.500	-	1.5
DT54-LEO5%	6.765	-	0.168	1.5
DT54-IBU-LEO5%	6.765	1.500	0.168	1.5

## Data Availability

The data presented in this study are available on request from the corresponding author.
